# Next Frontiers in Embedded Research Career Pathways; A Response to Recent Commentaries

**DOI:** 10.34172/ijhpm.9680

**Published:** 2025-12-27

**Authors:** Meghan McMahon, Bahar Kasaai, Rhonda Boateng, Nida Shahid, Richard H. Glazier

**Affiliations:** ^1^Canadian Institutes of Health Research, Institute of Health Services and Policy Research, Toronto, ON, Canada.; ^2^Institute of Health Policy, Management and Evaluation, University of Toronto, Toronto, ON, Canada.; ^3^Canadian Institutes of Health Research, Institute of Population and Public Health, Montréal, QC, Canada.; ^4^Institute of Clinical Evaluative Sciences, Toronto, ON, Canada.; ^5^MAP Centre for Urban Health Solutions, St. Michael’s Hospital, Toronto, ON, Canada.; ^6^University of Toronto Department of Family and Community Medicine, Toronto, ON, Canada.; ^7^Dalla Lana School of Public Health, University of Toronto, Toronto, ON, Canada.

 Our career pathways study tracked the early employment outcomes of the first three cohorts of embedded researchers in the Canadian Institutes of Health Research’s (CIHR’s) Health System Impact Program (HSIP).^1^ The HSIP was launched in 2017 with a clear mandate: to help PhD trainees and graduates in health services and policy research develop impact-oriented career readiness for a range of sectors and roles, within and beyond academia.^2^ We believed it was important to understand the extent to which the program was achieving this aim.

 Our results showed high post-fellowship employability that spanned sectors within and beyond the academy, and that fellowship alumni positively perceived the program’s success in elevating their career readiness and impact potential.^1^ We suggested these findings were an indication that the HSIP may be helping to equip fellows with the skills, readiness, and networks to contribute to a broad array of employment sectors and roles. Given the limited information available on the career pathways of doctoral graduates, we were excited to learn from the insightful commentaries submitted in response to our study.^3-8^

 Because the program was in its early stage at the time of analysis, alumni had only recently transitioned into the workforce. Therefore, our study focused on describing immediate post-fellowship career outcomes (their positions, types and sectors of employment organizations) and self-reported experiences and satisfaction about how the HSIP supported the fellows’ career readiness. Several of the commentaries emphasized the value of studying long-term career trajectories, transference of skills and experience in the workforce, and contributions and impacts in the embedded organizations.^3-6^ We firmly agree. These domains are understudied and the HSIP provides a unique and timely opportunity for evaluation and learning. Now in its eighth cohort year with 340 fellows, early career researchers (ECRs) and alum and over 140 health system and 25 academic partner organizations, the HSIP is committed to continuous learning and data-informed iterative improvement. Since publishing the career pathways study, the HSIP has undergone several enhancements that align with suggestions raised in the commentaries. We briefly describe a select few below.

 First, to support an embedded research career pathway, the program expanded its core streams in 2024 from doctoral and postdoctoral to include ECRs. The new Embedded ECR Award was designed using an evidence-informed and community-engaged process^9^ and builds on the core HSIP design elements (eg, embeddedness, dyad mentorship, protected academic time, enriched core competency professional development, training platform). Adaptations in the ECR design were intended to support career progression and sustainability, and include longer duration (four years, versus one and two years at the doctoral and postdoctoral levels, respectively), higher matched funding from the host organizations (50%, versus 30% at the doctoral and postdoctoral levels), and coaching and mentorship (versus supervision and mentorship at the doctoral and postdoctoral levels). By increasing the commitment from host academic and health system organizations (HSOs), the program aims to improve sustainability and retention of the ECR when the award funding ends. The first cohort of 12 ECRs was funded in 2024; time will tell whether this goal is achieved.

 Second, a new Health System Impact Training Platform provides dedicated training and supports to all HSIP interest-holders. Led by a pan-Canadian team of alum and mentors with lived experience in the HSIP, the Training Platform features customized offerings for: fellows and ECRs throughout their award; alumni as they progress in their careers (noted as essential by Embrett and Sim^3^); and mentors (noted by Wood and Daneshmand as critical^5^) and host partner organizations (identified as important by Yano^6^). The inaugural HSIP focused primarily on preparing high-quality fellows ready for system engagement and impact-oriented careers. The Training Platform builds on this focus to ensure mentors are also supported to help fellows navigate the opportunities and challenges of being an embedded researcher, and host partner organizations are supported to optimize their engagement with research and evidence-informed decision-making. The importance of training and supports for the embedding environments and preparing HSOs to be effective partners in research co-production and use has been identified elsewhere as critical for building high-performing ecosystems for embedded research.^6,10^

 Third, the health services and policy research core competency framework underpinning the HSIP^11^ underwent a refresh to ensure it stayed relevant.^12^ The refreshed core competencies maintain continuity with the inaugural framework while also introducing two new transversal domains: Equity, Diversity, Inclusion, Accessibility, and Anti-Oppression; and Indigenous Cultural Safety and Humility. These additions support fostering more inclusive and equitable leaders and health systems. The Training Platform has incorporated the refreshed framework within its curricular offerings – an important step given the evidence from the original competency framework^11^ that it is a mechanism for fellows to develop the full suite of enriched core competencies.^13,14^

 Across these program refinements, a commitment to continuous program learning and improvement has been maintained. Current improvement initiatives include updating alumni career profiles^15^; undertaking a multi-methods study of health system priorities and the embedded research methods used in practice (forthcoming); an impact analysis building on the initial embedded research impact casebook (forthcoming)^16^; and refreshing the alumni survey to track role types, career pathways, and employment outcomes (forthcoming).

 While these enhancements represent progress, for embedded researcher careers to flourish and sustain in complex systems facing fiscal constraint and competing priorities, there is work to be done across multiple fronts. Embrett and Sim suggest that embedded researcher position descriptions are needed that include an academic anchor position, opportunities for continuous professional development, and mentorship.^3^ They highlight that the formal partnerships between the academy and HSOs that are required during the HSIP fellowship may end afterward. This, they suggest, introduces risk to the organization’s ability to use rigorous evidence and to the embedded researcher’s academic network, research skills, and scientific integrity. A recent evidence and gap map of embedded researchers shows heterogeneity in embedded researcher models.^17^ There may be value for future work that provides clarity to the embedded researcher role, its relationship with the academy, the organizational culture and supports required for embedded research to thrive, and the impact metrics of success.

 Work is also needed to define, recognize, and reward embedded research impact. Academic outputs, like publications and grants, insufficiently capture embedded research impact, as noted by Lopatina et al.^7^ In the context of embedded research, impact encompasses contributions such as strengthening organizational research capability; advancing cultures of learning and improvement; building relationships with patients, clinicians and decision-makers to co-produce relevant evidence; and mobilizing evidence to inform decision-making.^7,18^ We support calls for an impact framework tailored to embedded research^7^– one that is endorsed by universities, research funders, and HSOs.

 Finally, while the potential for impact via embedded research is promising, the literature and lived experiences in the HSIP suggest there are structural tensions and paradoxes that warrant attention.^19^ Some of these include tensions between priority-driven research versus research independence, academic metrics for tenure and promotion versus policy and practice impacts in HSOs, the workload and expectations associated with a dual academic and health system affiliation, and being embedded in a research-ambivalent organizational culture.^19-21^

 The field of embedded research is growing in Canada and builds on a history of applied scholarship and integrated knowledge translation. It is gaining momentum as a knowledge mobilization strategy to align research with the priorities and evidence needs of HSOs and, in doing so, bridge the gap between research and action.^17,22^ In a health systems context of complex challenges, evolving health needs, and suboptimal performance, bridging the perennial gap between research and practice is urgently needed. Initiatives highlighted in the commentaries, including the CIHR HSIP,^2^ the Nova Scotia Network of Scholars,^8^ and the Ontario Health Team Impact Fellowship^4^ are building a cadre of emerging leaders with the interest, skills, and relationships to advance cultures of evidence use across sectors, organizations, and roles – cultures where research is key to improve health and system performance. Research funders, universities, and HSOs must now work together to support the longitudinal and sustained growth and impact of this field. The HSIP’s ongoing evaluation will continue to generate insights to inform this collective work.

## Acknowledgements

 The authors thank Ms. Erin Thompson, former member of the Institute of Health Services and Policy Research (IHSPR), for contributing towards the original career pathways study. The authors acknowledge and thank all of the HSIP awardees, mentors, alum, and partners for their leadership and contributions to embedded research. The authors also thank the commentary authors for their outstanding and insightful reflections on the study.

## Disclosure of artificial intelligence (AI) use

 Not applicable.

## Ethical issues

 Not applicable.

## Conflicts of interest

 Authors declare that they have no conflicts of interest.

## Disclosure

 The views expressed herein are solely those of the authors and do not necessarily reflect those of CIHR.
